# The Lapse

**Published:** 1871-08

**Authors:** T. L. Buckingham


### ARTICLE X.

### *The Lapse.*

By T. L. Buckingham, D. D. S.

In the January number of the *Dental Times* I published an article on "Reducing Gold and Silver Scraps to Plate." In giving a process to reduce the chloride of silver to a metallic state, I said that "the chlorlde should be covered with fresh water, add a small portion of sulphuric acid, then put a few small pieces of zinc in the vessel and let it stand for a day. *The chlorine will leave the silver and combine with the zinc.*" To what is stated in this last sentence one of the editors of the *Dental Register* takes exception, and thinks I have not correctly explained the changes that have taken place. His paragraph reads thus, "The closing sentence contains a lapse. The zinc decomposes the water, taking oxygen and liberating hydrogen. The hydrogen takes the chlorine from the silver. As the oxide of zinc is formed it unites with the sulphuric acid. The liquor contains sulphate of zinc, not chloride, and the escaping gas is hydrochloric acid, not hydrogen. And Professor B. knew all this as well as anybody, and he should have written more carefully in writing for the young, as he proposed to do." Now, the first part of this paragraph is partly correct, and I committed an error in not explaining the reaction more fully. "The zinc decomposes the water, taking oxygen and liberating hydrogen," and this is the first change that takes place. "The hydrogen takes the chlorine from the silver. As the oxide of zinc is formed it unites with the sulphuric acid," and the sulphate of zinc, being soluble, is dissolved in water. But what becomes of the hydrogen after it has taken the chlorine from the silver? The editor says the gas which escapes is hydrochloric acid (gas) and not hydrogen.

Here he commits a palpable error. He knows as well as anybody, and he should have written more carefully when he criticises for the young, that hydrochloric acid gas is one of the most soluble gases we have. Water at the ordinary temperature will take up nearly five hundred times its volume of this gas, so that if there was no more hydrogen liberated by the sulphuric acid acting on the oxide of zinc than the chlorine of the chloride of silver would combine with, there would be no gas escape; it would be all dissolved in the water, or until it became hydrochloric acid, as strong as we buy it in the shops. That gas does escape is evident from the effervescence that is seen while the process is going on.

The Dr. says it is "*not hydrogen.*" What is it? I contend that it is hydrogen and nothing else, and in order to verify this, I made several experiments. In an eight ounce bottle which I arranged with a cork and tube so that I could collect the gas, I put some chloride of silver, water, zinc and a little sulphuric acid, and as the gas passed over I collected it over water as we usually collect hydrogen gas. I then applied a lighted taper to the gas I had collected, and it ignited with an explosion at the mouth of the vessel, and when I passed the taper up into the gas, it was extinguished, and it reignited when I drew it out. I then introduced a glass tube drawn out at one end to a fine orifice, into the end of the gum tube through which the gas was passing, and ignited the gas at the small orifice, where it burned with all the appearance of hydrogen. From these results I concluded it was hydrogen. If it had been hydrochloric acid gas it would have been absorbed by the water, or if it had collected in any other way than over water it would not have burned.

In order to test whether chlorine was present, I passed some of the gas through a solution of nitrate of silver, and although the gas passed through for more than five minutes there was not the least indication of chlorine. To verify this experiment, I took a drop of hydrochloric acid on a glass rod, and put it into the nitrate of silver solution, when there was instantly formed a thick precipitate of the chloride of silver. From this I concluded that no chlorine came over with the hydrogen. As the gas that escapes is hydrogen, where does it come from? It has been shown that the hydrogen that is liberated by the zinc decomposing the water, goes to the chlorine of the silver and forms hydrochloric acid. The chlorine of this compound has a strong affinity for zinc, and combines with it even in the presence of sulphuric acid, so that in the chemical changes that are going on, both the sulphate and chloride of zinc are formed, and as they are both soluble they are retained in water. By the decomposition of the hydrochloric acid by the zinc, an additional quantity of hydrogen is set free, and from the two sources more hydrogen is generated than the chlorine of the silver requires to convert it into hydrochloric acid. The excess escapes in the form of gas.

If I am wrong in the above statements I hope the Doctor will correct me.—*Dental Times.*

